# Restored river habitat provides a natural spawning area for a critically endangered landlocked Atlantic salmon population

**DOI:** 10.1371/journal.pone.0232723

**Published:** 2020-05-21

**Authors:** Tuomas Leinonen, Jorma Piironen, Marja-Liisa Koljonen, Jarmo Koskiniemi, Antti Kause

**Affiliations:** 1 Natural Resources Institute Finland (Luke), Helsinki, Finland; 2 Organismal and Evolutionary Biology Research Program, Faculty of Biological and Environmental Sciences, University of Helsinki, Helsinki, Finland; 3 Natural Resources Institute Finland (Luke), Joensuu, Finland; 4 Department of Agricultural Sciences University of Helsinki, Helsinki, Finland; 5 Natural Resources Institute Finland (Luke), Jokioinen, Finland; Natural History Museum of London, UNITED KINGDOM

## Abstract

Supplementing endangered fish populations with captive bred individuals is a common practice in conservation management. The aim of supplementary releases from hatchery broodstocks is to maintain the viability of populations by maintaining their genetic diversity. Landlocked Lake Saimaa salmon (*Salmo salar* m. *sebago*) has been critically endangered for the past half-century. As a result of anthropogenic disturbance, especially construction of hydroelectric power plants, the Lake Saimaa salmon has become completely dependent on hatchery broodstock. Recently, habitat restoration has been done in one of the former spawning rivers with the aim of creating a new natural spawning ground for the critically endangered population. Hatchery fish releases have also been revised so that in addition to juveniles, adult fish from the hatchery and from the wild have been released into the restored river. We assessed here if a restored river stretch can be used as a natural spawning ground and juvenile production area with the aim of improving genetic diversity of the critically endangered Lake Saimaa salmon. By constructing a pedigree of the released adults, and juveniles sampled from the restored river, we found that the majority of the released adults had produced offspring in the river. We also found that wild-caught spawners that were released into the restored river had much higher reproductive success than hatchery-reared parents that were released into the restored river at the same time. We found no significant differences in genetic diversity between the parent and offspring generations. Meanwhile, relatedness among different groups of adults and juveniles varied a lot. For example, while the hatchery-reared females were on average half-siblings, wild-caught females showed no significant relatedness. This highlights the importance of using pedigree information in planning the conservation and management of endangered populations, especially when artificial propagation is involved.

## Introduction

The maintenance of genetic variation is essential to the survival of natural populations. Genetic variation enables populations to adapt to changing environmental conditions, and to retain their viability by reducing the negative impacts of inbreeding on individual survival and reproduction. To prevent the loss of genetic variation in endangered populations, a common practice has been to supplement them with captive bred individuals [1]. This strategy is especially common in the management of endangered fish populations (reviewed by [2]), many of which depend their existence entirely on restocking [3].

The ultimate goal of most conservation efforts is a genetically diverse, self-sustaining population in a natural environment [4], devoid of detrimental levels of inbreeding or introgression from non-native captive bred populations. There exists a delicate balance between supplementing natural populations with individuals from captive breeding programs to increase the effective population size, while maintaining the genetic variation of the natural population at a level that ensures the long-term viability of the population. It is thus imperative that the genetic structure of the natural populations, as well as the captive bred population used to supplement the natural population, is known, when aiming to establish self-sustaining populations in the wild. There are few examples of increased abundance of adult fish in the spawning grounds after hatchery stockings (e.g. [5–8]). Empirical evidence together with theoretical work suggests that for the increased abundance to be permanent, and the critically endangered populations to be re-established, measures to maintain genetic variation, such as replenishment of hatchery stocks with parents from the wild [9, 10], should be done in concert with habitat restoration [11, 12].

**Fig 1:**
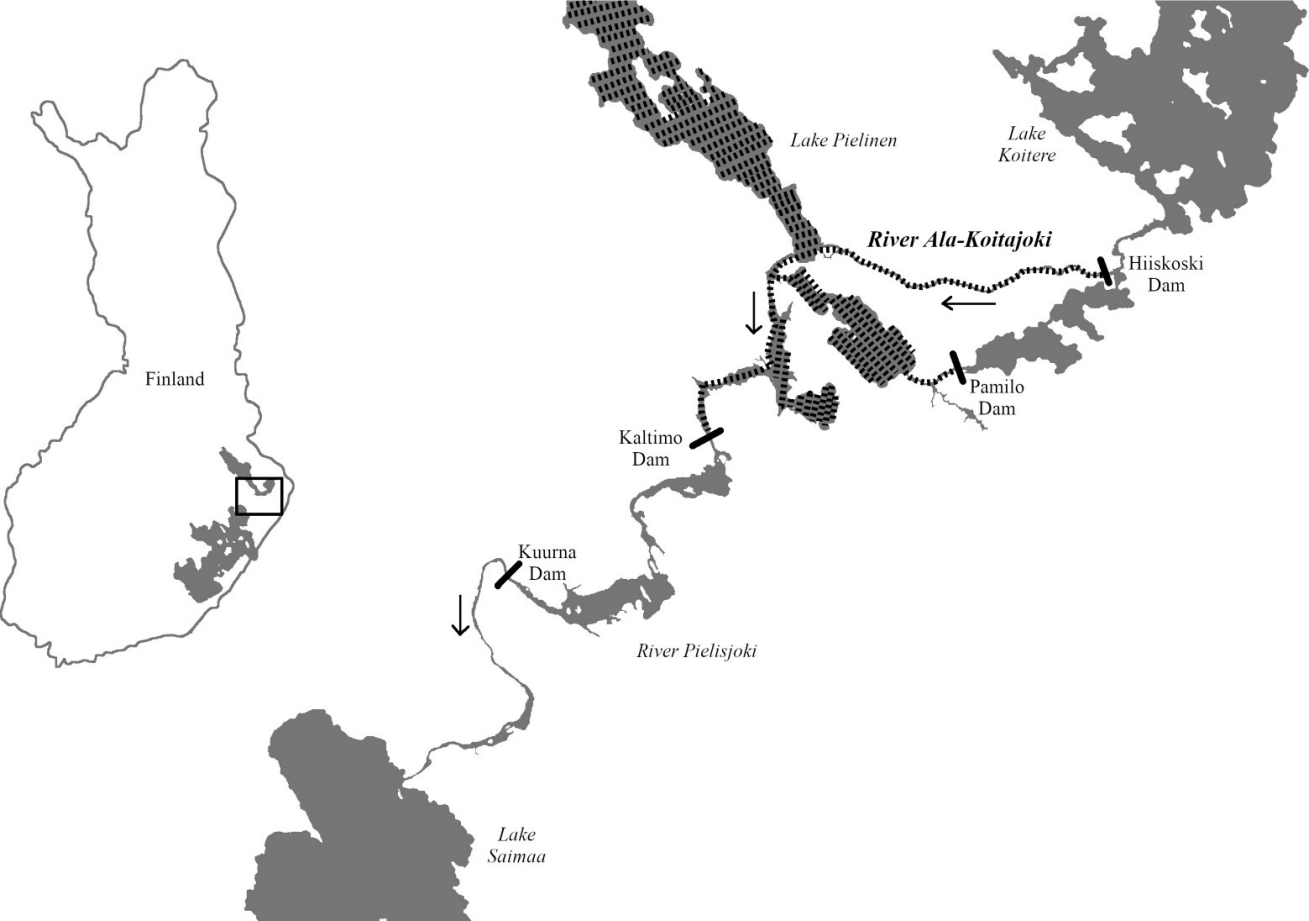
Map of the study area. The restored River Ala-Koitajoki with hydroelectric power plants (black bars), which cut off the upstream access of salmon in the river. The areas accessible to the salmon transferred into the restored River Ala-Koitajoki are marked with black shading. The direction of the river flow is marked with black arrows.

Atlantic salmon in the Finnish Lake Saimaa (*Salmo salar* m. *sebago*) became landlocked after the last glaciation ca. 10,000 years ago, and has since been isolated from anadromous salmon populations [13]. Following human influence, especially hydroelectric power plant construction in the mid-1900s, the landlocked salmon in Finland became virtually extinct in the wild, and has since been completely dependent on releases of hatchery-reared juveniles [14]. Although there may be isolated cases of natural reproduction, juvenile production relies almost completely on hatchery-reared broodstock. The hatchery-reared broodstocks are founded annually by catching ascending mature landlocked salmon below the Kuurna dam, which restricts access into the spawning areas in the rivers Pielisjoki and Ala-Koitajoki (Fig 1). The dams also directly contribute to a reduction in the viability of suitable spawning grounds due to changes in water levels and flow.

In 2013, a decision was made to try to re-establish the natural life cycle of the Lake Saimaa salmon. The plan was to restore the original spawning grounds and rapids in the River Ala-Koitajoki, which is cut off by two dams (at Kaltimo and Hiiskoski) from the juvenile-stocked river areas of River Pielisjoki and Lake Koitere (Fig 1). The aim was to make the River Ala-Koitajoki a natural spawning ground and juvenile reproduction area. Following the Supreme Administrative Court of Finland judgement in 2013, the water flow in the River Ala-Koitajoki was also increased from 2 to 5 m^3^/s to aid spawning ground restoration attempts. Because the spawners from Lake Saimaa do not have a direct access to the restored river stretch, a new stocking scheme was introduced, where mature salmon from the government hatchery in Enonkoski and mature fish grown in the wild in the River Pielisjoki were released into the restored river stretch of the river to spawn. At present, with the hydroelectric power plants still restricting fish movement, the plan involves relocating the smolts born in the restored river into the Lake Saimaa to continue their life cycle into mature adult stage.

Our main aim here was to find out whether the restored river stretch can function as a natural spawning and nursery area. This would be the case if the spawners brought to the restored river Ala-Koitajoki succeed to reproduce in the wild, and if the genetic diversity of the parental population is sufficiently transferred into their wild-born offspring generation. We tested this by 1) reconstructing a pedigree of the parents and offspring caught in the river Ala-Koitajoki, to find out how many of the parents have reproduced in the wild and if the reproductive output is evenly distributed among the parents, 2) estimating the relatedness among the parents and the offspring to find out whether the risk of inbreeding increases when reproduction takes place in a natural environment, and 3) comparing the indices of genetic divergence between the generation originating in hatchery (parents) and the generation originating in the wild (offspring).

## Materials and methods

### Samples

The parental generation fish were either raised in Enonkoski fish hatchery of the Natural Resources Institute Finland (Luke), or caught as adults in the wild in the River Pielisjoki, below the Kuurna and Kaltimo dams, which restrict salmon access to the River Ala-Koitajoki (Fig 1). The wild caught parents originated also from hatchery progeny of Enonkoski station, and had been released in the River Pielisjoki as two-year-old smolts. The parental generation fish were brought into the River Ala-Koitajoki (WGS84 coordinates: 62°51’29”, 30°31’44”) to spawn in their natural habitat at the age of 4–6 years. The mature fish were allowed to mate and spawn naturally without any human interference.

The hatchery-raised adults were released into the restored river in 2014 and 2015, and the wild-caught, lake-ranched, adults in 2014–2016. All the 122 potential parents were tissue-sampled with fin-clipping prior to their transfer into the River Ala-Koitajoki (Table 1). The offspring were tissue-sampled in the River Ala-Koitajoki in 2015–2018 at different life stages (eggs, parr, smolt; Table 1), according to corresponding year-classes. The fish were anaesthetized prior to fin clipping using clove oil (5mgL^-1^). In total, 650 offspring were sampled. Offspring sampling was done only in areas, where no juvenile releases had taken place to ensure that they were wild born. In addition, the offspring represented year-classes, which were not released. Salmon spawning nests were located by snorkeling in the spring before the onset of hatching. From each nest, 8–10 eggs were sampled, and each egg was treated as a separate sample.

**Table 1:** The number of Saimaa salmon sampled each year from each developmental stage.

Year	2014	2015	2016	2017	2018	Total
***Hatchery-reared adults***						
*Males-hatchery*	3	6	-	-	-	**9**
*Females-hatchery*	10	4	-	-	-	**14**
***Wild-caught adults***						
*Males-nature*	17	14	20	-	-	**51**
*Females-nature*	10	14	24	-	-	**48**
***Juveniles***						
*Smolts*	-	-	30	70	99	**199**
*Parr*	-	69	102	62	-	**233**
*Eggs*	-	60	48	110	-	**218**
**Total adults**	40	38	44	0	0	**122**
**Total juveniles**	0	129	180	242	99	**650**

‘Wild-caught adults’ were caught in the wild as adults and released into the restored river, while ‘hatchery-reared adults’ were born and raised in a hatchery, and then released into the river.

The study was carried out in accordance with the recommendations in the Association for the Study of Animal Behaviour (ASAB) guidelines for ethical treatment of animals and in compliance with Directive 2010/63/EU of the European Parliament and the Council of 22 September 2010 on the protection for animals used for scientific purposes. The protocols were approved by the Finnish Animal Experiment Board (license numbers ESAVI/5361/04.10.07/2013 and ESAVI/8190/04.10.07/2017). Permission for the field site access was provided by the landowner, Vattenfall AB.

### Microsatellite analysis

DNA was extracted with Qiagen DNeasy Blood&Tissue Kit from the roe (egg) samples, and DNeasy 96 Blood&Tissue Kit from the fin samples, using the ‘Animal Tissues’ protocols given in the kit manuals. The PCRs were done using Qiagen Type-it Microsatellite PCR Kit. For the DNA from fin samples, 15 μl reactions with 7,5 μl of kit master mix, and 4,5 μl of the extracted DNA were used. For the DNA from the egg samples, 25 μl reactions with 12,5 μl of kit master mix, and 7,5 μl of extracted DNA was used. 17 microsatellite loci were analyzed in three multiplex-reactions (S1 Table). The markers have been used in a number of earlier studies on genetic divergence and stock composition of the Atlantic salmon (e.g. [15, 16]), which allows for comparisons across studies. The same set of markers have also been used for all Baltic Sea salmon stock composition analyses in the EU-sampling program since 2006. This set of microsatellite markers was originally selected based on their roughly uniform distribution across the Atlantic salmon genome (see linkage map in [17]). The multiplexes, primer concentrations, and dyes are shown in S1 Table. The kit manual’s ‘Optimized cycling protocol for multiplex PCR amplification of microsatellites’ was used with the annealing temperature of 56°C. Microsatellite genotypes were detected with an Applied Biosystems ABI 3130 automated DNA sequencer, and analysed with GeneMapper analysis software v5.0, with the size standard of Applied Biosystems GeneScan 500LIZ. Automatic outputs were checked for errors and corrected manually.

Deviations from Hardy-Weinberg equilibrium (HWE) for each locus and sampling group (hatchery- reared adults, wild-caught adults, eggs and juveniles grouped by the developmental stage and sampling year; S1 Table) combination were tested with Fisher’s exact test, and linkage disequilibrium (LD) between pairs of loci in each group was tested using the log likelihood ratio statistic. The default Markov-chain parameters (1000 dememorization steps, 100 batches, and 1000 iterations per batch) in Genepop v4.2 [18] were used for both tests. Significance levels (at p<0.05) for the HWE and linkage disequilibrium tests were adjusted for multiple comparisons using the sequential Bonferroni method [19]. Presence of null alleles, observed (*H*_*O*_) and expected (*H*_*E*_) heterozygosity, and inbreeding coefficient (*F*_*IS*_) at each locus were tested using PopGenReport R package v3.0.4 [20].

Two of the loci showed no polymorphism (*SSa85* and *SSsp3016*), and therefore genotypes from 15 microsatellite loci were used in subsequent analyses (S1 Table). One of the loci (*SSsp2210*) showed polymorphism only in the wild-caught adults, and parr from 2015 and 2016 (S2 Table). The results with and without this locus did not differ qualitatively (data not shown), so all the presented results are based on 15 microsatellite loci. Most of the groups and loci were in Hardy-Weinberg equilibrium: only six of the 165 tests across all loci by all groups differed significantly from the equilibrium after Bonferroni correction (S2 Table). There was also little evidence of linkage disequilibrium: 34 pairs of loci across the 1155 combinations of pairs of loci and groups (2.9%) showed significant linkage disequilibrium test values. None of the loci showed evidence of null alleles (S1 Table).

### Pedigree reconstruction

Pedigree reconstruction was done to find out how large a proportion of adults had managed to reproduce, and whether the reproductive output was evenly distributed among the parents. The pedigree was reconstructed by assigning parentage and sibships with full likelihood as implemented in the program Colony2 [21]. The parentage and sibship assignments were done separately for three different cohorts: adults released in 2014 and their potential offspring, adults released in 2015 and their possible offspring, and adults released in 2016 and their possible offspring (Table 2). This was done to reduce computation time (by excluding ‘impossible’ parent-offspring relationships e.g. adults from 2016 with offspring from 2015). The full likelihood assignments were estimated with medium length runs with high precision without a sibship prior, and with sibship size scaling (to prevent possible false reconstruction of large sibships into two or more full sibships [22]). Three replicate runs were done with three different genotyping error estimates (1: Allelic dropout rate, AD = 0 and other typing error, GE = 0.001; 2: AD = 0.001 and GE = 0.001; 3: AD = 0.001 and GE = 0.0001). If there were inconsistencies among different runs, the parent assignments where at least half (5/9) of the runs produced the same outcome, were taken as correct. In total, 16% of all parent-offspring assignments were not in total agreement in all nine runs. To get an idea of the genetic contribution of the parents to the offspring generation, the number and size of full-sib families were counted from the same Colony2 runs that were used for the pedigree reconstruction. Only full-sib families with an inclusion probability of higher than 0.8 were included in the results. The inclusion probability describing the extent to which each family is splitable–the lower the probability, the higher the likelihood that the family can be split into two or more families [21].

**Table 2:** The three cohorts used in the pedigree estimation.

	Year class of parents		
*Offspring sampled as*:	2014	2015	2016
*Eggs*	2015	2016	2017
*Parr 0+*	2015	2016	2017
*Parr 1+*	2016	2017	-
*Smolts 2+*	2017	2018	-
Number of possible offspring	462	295	172

Each column shows the sampling and release year of the parents together with the sampling year and age class of their possible offspring. The total number of possible offspring for each year class of parents is displayed on the bottom row.

### Relatedness estimation

Relatedness estimates among the parents and the offspring were calculated to find out whether reproduction in a natural environment increases the risk of inbreeding in future generations. The mean relatedness was estimated for several subgroups. First, the mean relatedness was estimated among the adults and among the juveniles, then mean relatedness was estimated for every annual sample and age group of juveniles, and finally mean relatedness was estimated for each annual adult cohort (Table 2). Because reproductive success can be different between sexes (e.g. [5]), we also estimated relatedness separately for both sexes. Relatedness among individuals within the groups was calculated using Wang’s relatedness estimator [23] with correction for sample size as implemented in the demeRelate package for R [24]. The logic of the relatedness test implemented in demeRelate is that a threshold value for a proportion of first and second order relatives (full- and half-sibs; relatedness = 0.5 and 0.25, respectively) is calculated from a randomly drawn group of similar size as the group under study using logistic regression. *Χ*^*2*^ probability test is then used to assess whether the focal group contains more full- and half-siblings than expected in the randomly drawn group of similar size. Here, the thresholds for full- and half-siblings were calculated with 1000 simulated pairs and 1000 randomizations.

### Genetic diversity

Estimates of genetic diversity were calculated to find out whether the genetic diversity present in the parental generation is passed on to the offspring generation born in the natural environment. Estimates of heterozygosity and allelic richness were calculated for each sub-group (hatchery-reared and wild-caught adults, offspring grouped by sampling year and age) as well as for the parental generation and for the offspring overall. Locus-specific and average gene diversities, expected heterozygosity (H_E_; [25]), allelic richness, and allele frequencies were estimated using diveRsity R package v 1.9.90 [26]. Confidence intervals for the inbreeding coefficient (*F*_*IS*_) were calculated from 1000 bootstrap iterations.

The extent of genetic diversity among the parental generation and among the offspring was also measured by using effective population size (*N*_*e*_) estimates. Pedigree N_e_ was estimated with the sibship assignment method as implemented in Colony2 [21]. In the sibship assignment method, the frequencies of full and half sib dyads are used to estimate the current N_e_ [27]. The *N*_*e*_ estimates were calculated separately for each of the three adult cohorts and their potential offspring as well as for all the adults across the three cohorts combined, and all the juveniles across the three cohorts combined. N_e_ was also estimated for the whole population of genotyped individuals. Six replicate runs were done in Colony2 with parameters as above. Because very little differences were found in earlier runs with different genotyping error estimates, AD = 0.01, and GE = 0.01 was used in all replicate runs for the *N*_*e*_ estimation. In general, there were no large differences in N_e_ estimates among replicate runs with any chosen group of individuals (S3 Table).

## Results

### Distribution of reproductive output

The pedigree revealed that overall, a little more than half (53%) of the released adults had reproduced in the restored river. The proportion of adults, whose offspring was found in the restored river was much higher for the adults caught in the wild and brought to the River Ala-Koitajoki to spawn than for the adults raised in a hatchery until maturity and brought to the river to spawn (59/85 and 6/37 respectively; Table 3). The total numbers of offspring were also clearly higher for the wild-caught parents (n = 866) than for the hatchery-reared parents (n = 39). All the offspring of the hatchery-reared parents were from a mating with a wild-caught parent. The majority of the sampled offspring (87%) had their parents among the sampled adults (Table 4). Most of the offspring with unknown parents were smolts from 2017, who could have migrated into the sampling areas from the upper reaches of the river, where hatchery-reared juveniles are also released (Table 4).

**Table 3:** Distribution of reproductive output of Lake Saimaa salmon.

Parent group	Number of parents with assigned offspring	Total N parents	Proportion of parents with assigned offspring	Total number of offspring
*Hatchery-reared*				
Sires 2014	2	17	0.12	23
Dams 2014	1	10	0.1	1
Sires 2015	0	6	0	0
Dams 2015	3	4	0.75	15
*Wild-caught*				
Sires 2014	3	3	1	114
Dams 2014	5	10	0.5	147
Sires 2015	13	14	0.93	183
Dams 2015	9	14	0.64	143
Sires 2016	14	20	0.7	111
Dams 2016	15	24	0.63	168
*Sires total*	*32*	*60*	*0*.*53*	*431*
*Dams total*	*33*	*62*	*0*.*53*	*474*
*Hatchery-reared total*	*6*	*37*	*0*.*16*	*39*
*Wild-caught total*	*59*	*85*	*0*.*69*	*866*
**Total**	**65**	**122**	**0.53**	**905**

Dams and sires are divided into groups according to the year they were brought into the restored river, and whether they were reared in the hatchery or caught in the wild. Shown are the numbers of parents with offspring in each group, the total number of parents in each group, and the proportion of parents with offspring in each group.

**Table 4:** Assignment of parents to the offspring.

Offspring group	Total N	Number of individuals with no assigned parents	Number of individuals assigned to a sire only	Number of individuals assigned to a dam only	Proportion of individuals without assigned parents	Proportion of individuals with only one assigned parent
Parr 2015	70	7	2	11	0.10	0.19
Parr 2016	103	5	10	14	0.05	0.23
Parr 2017	62	1	2	22	0.02	0.39
Smolts 2017	70	56	4	2	0.80	0.09
Smolts 2018	101	16	33	9	0.16	0.42
Hiiskoski Eggs 2015	30	2	0	0	0.07	0.00
Kuusamonkoski Eggs 2015	30	1	2	1	0.03	0.10
Hiiskoski Eggs 2016	28	1	1	7	0.04	0.29
Kuusamonkoski Eggs 2017	10	0	0	2	0.00	0.20
Pamilonkoski Eggs 2017	50	0	7	0	0.00	0.14
Eggs All	148	4	10	10	0.03	0.14
**Total**	**702**	**93**	**71**	**78**	**0.13**	**0.21**

The table shows the number of offspring whose parents were not found among the sampled adults, the number of offspring with only one parent among the sampled adults, and their proportions of the total number of offspring. The offspring are divided according to their developmental stage and sampling year. The egg samples are further divided into sub-groups according to the place and year they were sampled.

**Fig 2:**
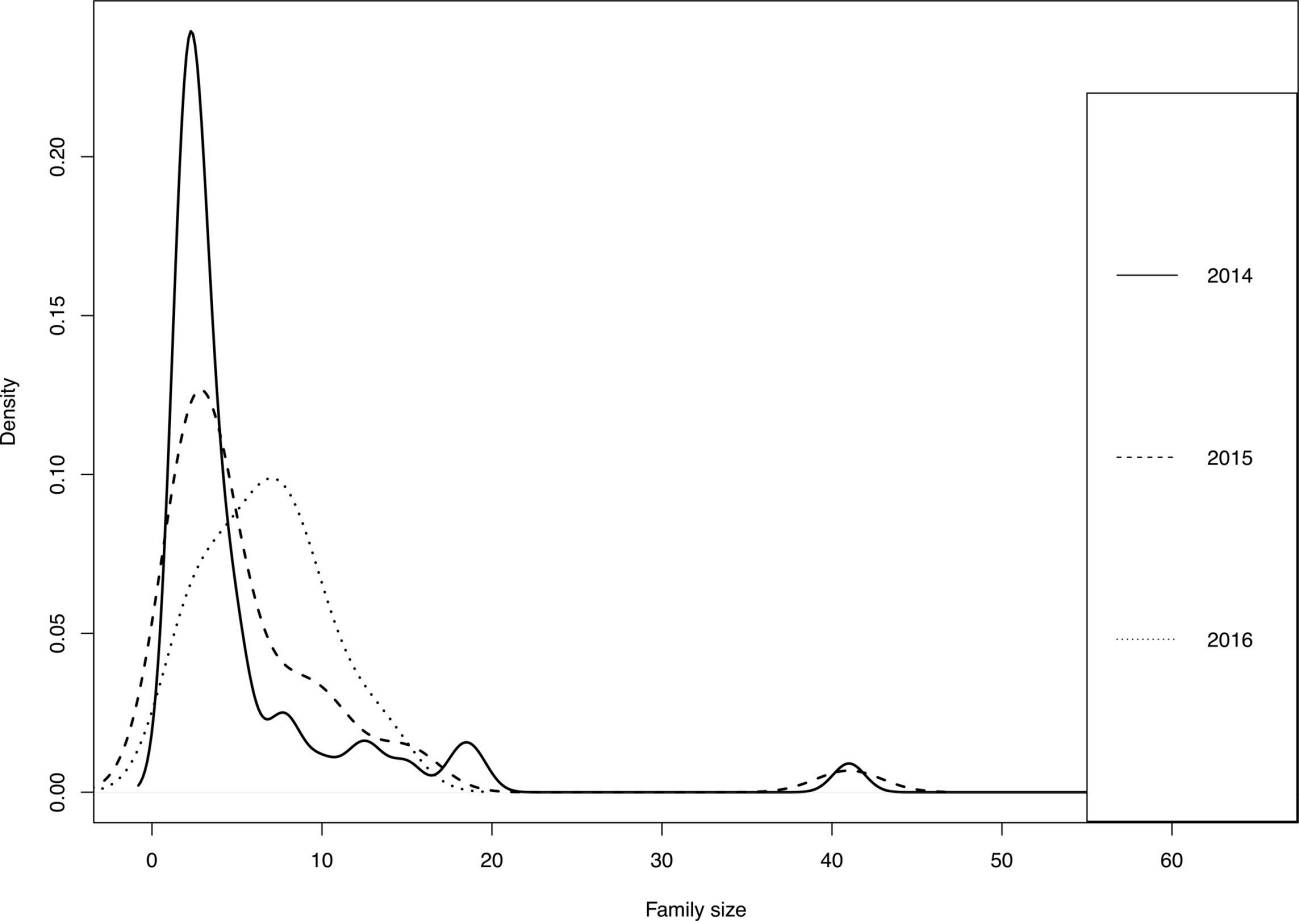
Full sib family sizes. Distribution of full sib families of different size in each yearly cohort of parents. (Smoothing of the distributions is based on Kernel density with bandwidths chosen by Silverman’s [28] rule of thumb).

Among the fish that managed to produce offspring, the mean number of mates was similar for dams and sires (1.70 and 1.76 respectively, both with range: 1–4) with relatively little variance (dams: 0.77 and sires: 0.98). The mean number of offspring produced by dams was 11.6 (range: 1–62, variance: 178), and by sires 12.2 (range: 1–70, variance: 247). The number of full-sib families ranged from 47 in 2014 to 16 in 2016 (Fig 2). The higher number of full-sib families in older cohorts most likely reflects the larger sample size of possible offspring in the older cohorts (Table 2). There were no large deviations in the numbers of offspring in each full-sib family, with the exception of the 2014 cohort, in which there were two relatively large families (Fig 2).

### Relatedness

Overall, relatedness was higher among the hatchery-reared adults than among the wild-caught adults or among the offspring (Tables 5 and 6). With the exception of males released in 2014, among which the relatedness estimate was lower (wang_xy_ = 0.05). The hatchery-reared adults were essentially full-sibs on average (Table 5). However, the overall observed number of full- and half sibs did not deviate significantly from a randomly drawn population, which reflects the low sample size in each yearly cohort of hatchery-reared adults (Table 5). Among the wild-caught adults, the only cohort, where the observed frequency of full-siblings was significantly lower than the expected frequency of full-siblings, were the females released in 2014 (Table 5).

**Table 5:** Relatedness among the adults released into the restored river.

Group	Sibship	Observed	Expected	X^2^	p (X^2^)	Relatedness (Wang)
Hatchery - Females 2014						0.23
	FS	0.31	0.07	7.25	**0.01**	
	FS+HS	0.64	0.31	8.73	**0**	
Hatchery - Males 2014						0.05
	FS	0.07	0.04	0.28	0.60	
	FS+HS	0.54	0.30	0.60	0.44	
Hatchery - Females 2015						0.19
	FS	0.17	0.00	0.00	1	
	FS+HS	0.83	0.17	3.00	0.08	
Hatchery - Males 2015						0.17
	FS	0.13	0.00	0.54	0.46	
	FS+HS	0.60	0.20	3.47	0.06	
Parents - hatchery						0.10
	FS	0.10	0.04	19.27	**<0.001**	
	FS+HS	0.41	0.26	33.75	**<0.001**	
Wild - Females 2014						-0.09
	FS	0.02	0.04	0.00	1	
	FS+HS	0.22	0.44	4.05	**0.04**	
Wild - Males 2014						-0.15
	FS	0.00	0.00	NA	NA	
	FS+HS	0.00	0.33	0.00	1	
Wild - Females 2015						-0.06
	FS	0.01	0.04	0.82	0.36	
	FS+HS	0.18	0.31	3.63	0.06	
Wild - Males 2015						0.03
	FS	0.03	0.04	0.00	1	
	FS+HS	0.32	0.29	0.10	0.75	
Wild - Females 2016						0.03
	FS	0.04	0.03	0.52	0.47	
	FS+HS	0.33	0.27	2.21	0.14	
Wild - Males 2016						0.02
	FS	0.05	0.05	0.00	1	
	FS+HS	0.28	0.31	0.20	0.65	
Parents - Wild						0.01
	FS	0.03	0.03	1.50	0.221	
	FS+HS	0.25	0.27	2.45	0.117	
Parents all						0.03
	FS	0.04	0.03	12.74	**<0.001**	
	FS+HS	0.30	0.27	18.14	**<0.001**	

The number of full-siblings (FS), and full- and half-siblings (FS+HS) in a random sample of unrelated individuals (expected) were compared to the *observed* number of full- and half-sibs in each group (see text for details) with a *Χ*^*2*^ test (with df = 1).

**Table 6:** Relatedness among the juveniles in the restored river.

Group	Sibship	Observed	Expected	X^2^	p (X^2^)	Relatedness (Wang)
Eggs 2015						0.13
	FS	0.19	0.04	217.50	**<0.001**	
	FS+HS	0.45	0.27	114.23	**<0.001**	
Eggs 2016						0.15
	FS	0.20	0.03	159.72	**<0.001**	
	FS+HS	0.50	0.28	108.98	**<0.001**	
Eggs 2017						0.04
	FS	0.09	0.04	137.17	**<0.001**	
	FS+HS	0.34	0.27	53.85	**<0.001**	
Parr 2015						0.22
	FS	0.27	0.04	496.86	**<0.001**	
	FS+HS	0.61	0.25	613.68	**<0.001**	
Parr 2016						0.04
	FS	0.12	0.04	261.18	**<0.001**	
	FS+HS	0.33	0.27	45.98	**<0.001**	
Parr 2017						0.00
	FS	0.06	0.04	5.55	**0.019**	
	FS+HS	0.25	0.28	4.78	**0.029**	
Smolts 2017						-0.01
	FS	0.04	0.03	3.53	0.06	
	FS+HS	0.26	0.27	1.03	0.311	
Smolts 2018						0.00
	FS	0.06	0.03	32.72	**<0.001**	
	FS+HS	0.26	0.25	2.28	0.131	
Offspring all						0.05
	FS	0.04	0.03	340.87	**<0.001**	
	FS+HS	0.26	0.28	219.36	**<0.001**	

The number of full-siblings (FS), and full- and half-siblings (FS+HS) in a random sample of unrelated individuals (expected) were compared to the *observed* number of full- and half-sibs in each group (see text for details) with a Χ^2^ test (with df = 1).

Among the offspring year-classes, the highest relatedness was found among the 2015 parr, who were on average half-siblings (wang_xy_ = 0.22; Table 6). The eggs from 2015 and 2016 were also relatively highly related, at a level similar to full cousins (wang_xy_ = 0.13 and 0.15; Table 6). Contrary to the parental generation, all the offspring groups had higher than expected proportion of full- and half-sibs in the group than a randomly drawn similar sized group of individuals–with the exception of 2017 and 2018 smolts (Table 6).

### Genetic diversity

The patterns of relatedness were also reflected in the estimates of inbreeding: the parental generation had a significantly lower *F*_*IS*_ than expected, while the offspring had a significantly higher *F*_*IS*_ than expected from a population at HWE (Table 7). Looking at the *F*_*IS*_ values in more detail, the groups with *F*_*IS*_ diverging from expectation were the hatchery-reared adults, the 2015 parr, and 2015 and 2016 eggs, all with a significantly negative *F*_*IS*_ (Table 7).

**Table 7:** Genetic diversity of the Lake Saimaa salmon samples in the River Ala-Koitajoki.

Group	N	H_E_	H_O_	N Alleles	Proportion of alleles	Allelic richness (95%CIs)		F_IS_ (95%CIs)	
Parents - hatchery	37	0.50	0.55	64	80.19	4.02	(3.73;4.20)	**-0.11**	**(-0.18;-0.07)**
Parents - nature	85	0.55	0.56	74	92.00	4.36	(4.07;4.60)	-0.02	(-0.06;0.02)
Eggs 2015	60	0.51	0.55	60	77.65	3.69	(3.47;3.93)	**-0.07**	**(-0.13;-0.02)**
Eggs 2016	48	0.48	0.51	53	71.08	3.38	(3.20;3.53)	**-0.06**	**(-0.11;-0.01)**
Eggs 2017	110	0.52	0.54	65	84.41	4.05	(3.80;4.27)	-0.02	(-0.06;0.01)
Parr 2015	69	0.46	0.51	61	80.51	3.58	(3.20;3.87)	**-0.10**	**(-0.15;-0.06)**
Parr 2016	102	0.51	0.50	66	80.92	3.85	(3.53;4.13)	0.02	(-0.02;0.05)
Parr 2017	62	0.53	0.52	71	89.24	4.25	(4.00;4.53)	0.01	(-0.04;0.05)
Smolts 2016	30	0.49	0.46	60	78.64	3.76	(3.47;4.00)	0.05	(-0.04;0.11)
Smolts 2017	70	0.56	0.56	72	92.32	4.30	(4.00;4.53)	-0.02	(-0.06;0.02)
Smolts 2018	99	0.54	0.55	70	88.13	4.27	(4.00;4.53)	-0.01	(-0.05;0.03)
Parents overall	122	0.54		75	92.48	4.85	(4.67;5.00)	**-0.04**	**(-0.07;-0.01)**
Offspring overall	650	0.54		81	99.52	4.92	(4.67;5.00)	**0.02**	**(0.01–0.04)**
**Total**	**772**			**85**	**100.00**				
**Mean**		**0.51**	**0.53**			**3.96**		**-0.03**	

The samples are grouped by the sampling year (parr and smolts) or the environment from where they were transferred (parents). Genetic diversity is expressed as expected (*H*_*E*_) and observed (*H*_*O*_) heterozygosity, number of alleles (N alleles), percentage of total alleles observed per locus in each group of samples (Proportion of alleles), allelic richness per locus per sample group with its corresponding 95% confidence intervals (Allelic richness), and global *F*_*IS*_ values for each sample with their 95% confidence intervals (*F*_*IS*_). *F*_*IS*_ values significantly different from zero are marked in bold.

There were no significant differences in the estimates of heterozygosity or allelic richness between the parental generation and the offspring generation (Table 7). Among the groups of offspring, there was a trend that the more recently sampled parr and smolts had a significantly higher allelic richness than the earliest samples [2015 parr: *A*_*R*_ = 3.58 (95%CI: 3.20–3.87) vs 2017 parr: *A*_*R*_ = 4.25 (95%CI: 4.00–4.53) and 2016 smolts: *A*_*R*_ = 3.76 (95%CI: 3.47–4.00) vs 2018 smolts: *A*_*R*_ = 4.27 (95%CI: 4.00–4.53)]. This trend was also reflected in the higher values of heterozygosity in the more recent samples (Table 7).

Possible changes in the extent of genetic diversity among generations were also measured with *N*_*e*_ estimates. There were no significant differences in the overall *N*_*e*_ between parental and offspring generations judging by the overlap of the 95% confidence intervals (Table 8). The *N*_*e*_ tended to be lower in the 2016 juveniles than in their potential parents, reflecting the lower number of possible offspring when compared to the two earlier cohorts, 2014 and 2015 (the number of possible offspring of the 2014 parents = 462; 2015 = 295, 2016 = 172; Table 2).

**Table 8:** Effective population size.

		Parents			Offspring		All		
Year	n	N_e_	(95%CI)	n	N_e_	(95%CI)	n	N_e_	(95%CI)
2014	40	41	(26–69)	462	49	(34–79)	502	40	(17–68)
2015	38	44	(27–76)	295	45	(31–69)	333	39	(25–66)
2016	44	47	(29–75)	172	28	(17–48)	261	27	(17–62)
**Total**		**85**	**(62–116)**		**67**	**(48–93)**		**63**	**(45–93)**

Effective population size (*N*_*e*_) with the associated 95% confidence intervals and the sample sizes (n) in the three cohorts of Lake Saimaa salmon. Each cohort includes the parents stocked that year and their possible offspring.

## Discussion

The main aim of our work was to find out whether a restored river habitat can act as a natural spawning and reproductive area for a critically endangered landlocked salmon population that has no other natural spawning habitats left. We also wanted to find out, whether using the restored river for spawning and reproduction, instead of stockings based solely on hatchery-reared individuals, could provide a solution for improving the genetic diversity of the landlocked salmon. Our results show that natural reproduction is possible in the restored river habitat. Majority of adults released into the restored river stretch produced offspring, although the wild-caught parents were more successful in producing offspring than the hatchery-reared adults. There were also no signs that genetic diversity or the effective population size would be lower among the offspring than among the parental generation.

### Successful reproduction in the restored river revealed by pedigree

Accumulating evidence indicates that the key to successful conservation of endangered fish populations is preservation and restoration of suitable habitats for spawning, rather than solely increasing the number of released individuals [11]. Another key issue is the origin of the released individuals. Whenever possible, these should be selected from wild strains [2, 10]. In line with scientific evidence, many countries have adopted policies where habitat restoration is the primary means for conserving endangered salmon populations, and only when necessary, it is combined with releases of juveniles originating from hatchery crosses using wild broodfish [29, 30]. In the River Ala-Koitajoki, spawning habitats were restored and water flow was increased to create a new natural spawning and reproduction area for the landlocked Atlantic salmon population, where transferred mature fish could spawn naturally. The mature fish transferred into the restored river included both, hatchery-reared fish, which had spent their entire life-cycle up to the transfer in a hatchery, and wild-caught fish, which had been released into the wild as juvenile and thus spent the years up to maturation in a natural habitat.

Using restored river stretches as spawning habitats to manage endangered salmon populations has a number of advantages over supplementary stocking of hatchery-reared individuals. Hatchery-reared salmonids released into the wild have been shown to be inferior in fitness and reproductive success when compared to wild individuals (e.g. [31–34]), even when the period spent in captivity is short [35–37]. Our results also show that the parents reared in hatchery until maturity are less successful in producing offspring than the parents who were released to nature as juveniles. Several reasons have been proposed to explain this. First, fish have been shown to adapt genetically to hatchery conditions (reviewed by [38]). Second, there may be additional unintentional selection on inherited traits if stocked or artificially bred individuals are selected non-randomly [29, 35]. Random selection of breeding pairs in hatcheries may also lead to reduced fitness of offspring, due to the lack of sexual selection, which in nature increases offspring viability [33, 39, 40]. In a restored river habitat, the selective regime is close to natural, which should reduce the risks associated with hatchery adaptation, and lead to increased fitness of the future generations. In addition, mating in the restored river takes place naturally, meaning that the reproducing fish are subjected to sexual selection, which should further increase the viability of the offspring. However, allowing free mate choice in small populations entails risks because it might lead to a decrease in *N*_*e*_ and increase in inbreeding [39]. In the restored River Ala-Koitajoki, we found no reduction in *N*_*e*_ between the parental and offspring generations. Furthermore, the majority of stocked adults (53% of all adults, 69% of the wild-caught adults) were found to have offspring. The individual reproductive success was similar to earlier studies on anadromous Atlantic salmon (*S*. *salar*) populations in natural and semi-natural environments, that have used a similar approach of measuring reproductive success as the number of young produced [41–43]. Our results are also consistent with the earlier studies on natural Atlantic salmon: the variation in reproductive success in our study was high for both sexes [41, 42]. However, the number of mates was lower (mean 1.7, range 1–4 in both sexes) than what has been reported in natural Atlantic salmon populations [41–45]. The lower number of mates in our study population is likely due to the lower number potential mates (range: 3–24), when compared to the natural populations. Also, in the breeding population here, there were no young precocious males, which in wild anadromous Atlantic salmon populations increase the number of mates.

### The effect of natural reproduction on genetic diversity

There is a growing number of encouraging examples, where anadromous Atlantic salmon have started to return to the restored rivers, where they have been previously extirpated (e.g. [30, 46–49]). The common denominator in all the successful cases of re-establishment of anadromous salmon is the restoration of the formerly uninhabitable habitats. Apart from habitat restoration, there is a lot of variation in the factors that contribute to the success, or failure, of the attempts to restore extirpated populations, especially when the re-establishment relies on hatchery broodstock [1]. One of the main challenges in supplementary stocking of endangered populations involves the maintenance of genetic diversity. Populations subjected to natural and sexual selection in the wild, like the fish breeding in the restored River Ala-Koitajoki, are under the risk of losing genetic diversity at the expense of fitness. Among the hatchery-reared parents in our study there were more full- and half-siblings than should be expected from a random sample of similar size, which is a direct consequence of the low number of adults in each hatchery year class. The higher than expected relatedness leads to risk of inbreeding in the successive generations. However, among the hatchery-reared adults, the inbreeding coefficient, *F*_*IS*_, was negative, indicating a higher than expected number of heterozygotes for a population in Hardy-Weinberg equilibrium. The high relatedness combined with a negative *F*_*IS*_ is likely due to the hatchery population being established from a combination of different groups of fish originating from different areas of the Lake Saimaa, and from different year classes–the management strategy used to avoid inbreeding in controlled matings in hatcheries. The wild-caught parents did not have higher than expected relatedness or a *F*_*IS*_ different from zero. Based on these differences, careful management of the genetic background of adults in future stockings should be implemented. For instance, the stocked individuals should be selected so that the rates of loss of genetic variation and levels of inbreeding in future generations can be kept to a minimum. This is possible when the pedigree of the fish is known. Genetic management based on a known pedigree should also ultimately help to resolve the possible trade-offs in conserving genetic variation versus fitness [50].

**Fig 3:**
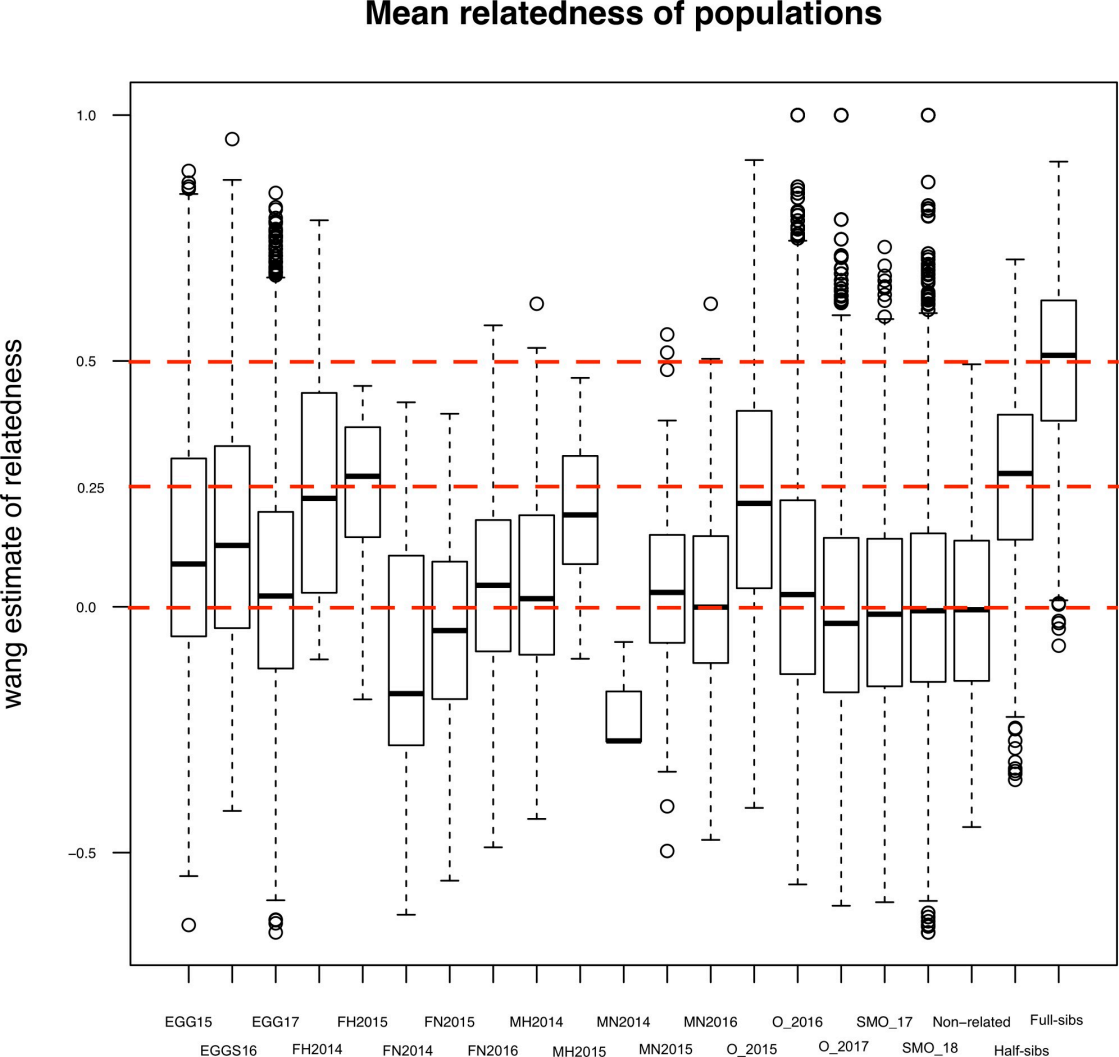
Relatedness estimates. Relatedness estimates [23] between pairs of individuals in the Lake Saimaa salmon divided according to sampling year (or release year of parents) and life stage. (solid lines: median, boxes: interquartile range; FH: hatchery-reared females, FN wild-caught females, MH: hatchery-reared males, MN: wild-caught males, O: parr, SMO: smolts).

Among the offspring there were more 1° and 2° relatives than should be expected in a random sample of similar size. However, there was a lot of variation in mean relatedness. The highest relatedness was among the 2015 parr that were half-sibs on average and eggs from 2015 and 2016 that were first cousins on average. The high relatedness among the eggs is understandable considering that each nest is likely to have been fertilized by a single male. The high relatedness among the 2015 parr is likely due to the majority of the sample belonging to a single large full-sib family (results not shown). Again, despite the high relatedness, the inbreeding coefficient, *F*_*IS*_ was significantly negative, possibly reflecting the mixed origin of the parents. While the mean relatedness estimates give an indication of possible problems to maintenance of genetic variation, from a management perspective it is more relevant to look the distribution of relatedness estimates. Although the mean relatedness is mostly relatively low, there is a lot of variance among pairwise relatedness estimates (Fig 3). This underlines the importance of considering the genetic structure of population when managing critically endangered populations. When a pedigree is available that covers most of the population structure, as in the case of the River Ala-Koitajoki salmon population, genetic variation can be maintained and even increased by careful selection of the fish used in future juvenile releases. Careful genetic management using the information from the pedigree should be the next step towards making the endangered Saimaa salmon population genetically sustainable, especially if done in concert with habitat restoration, water flow increase, and hopefully removal of all barriers to fish migration in the spawning rivers.

## Conclusions

In conclusion, our results show that the restored river habitat can function as a natural spawning and reproduction habitat for the critically endangered Lake Saimaa salmon, even when the spawners are transported from elsewhere. Transfer of adults into the restored river has resulted in wild-born offspring, providing a possible solution for the maintenance of genetic diversity and natural reproductive traits in the critically endangered Lake Saimaa salmon. The adults transferred from a hatchery had a clearly lower spawning success than the adults that were caught in the wild. This together with the large variance in the between-individual relatedness estimates highlights the importance of using pedigree information when planning the breeding of broodstock for an endangered population. Although it is premature to evaluate the future success of Ala-Koitajoki spawning population, the results indicate that a combination of genetic management of the hatchery broodstock and natural reproduction in the restored habitat could provide a sustainable solution for the preservation of the critically endangered Lake Saimaa salmon.

## Supporting information

S1 Table Microsatellite loci used for the analyses. (DOCX)

S2 Table Deviation from from the HWE for each locus and sampling group combination. (DOCX)

S3 Table Consistency of the estimates of effective population size. (DOCX)
