# Supplementary material for: Restored river habitat provides a natural spawning area for a critically endangered landlocked Atlantic salmon population
Source: PLoS One. 2020 May 21;15(5):e0232723. doi: 10.1371/journal.pone.0232723 (PMC7241772; doi:10.1371/journal.pone.0232723)
Supplement: S1 Table — (DOCX) [file pone.0232723.s001.docx]

**S1 Table.** **Microsatellite loci used for the analyses.**

AR – Allelic richness, He – expected heterozygosity, HO – observed heterozygosity
