# Supplementary material for: Restored river habitat provides a natural spawning area for a critically endangered landlocked Atlantic salmon population
Source: PLoS One. 2020 May 21;15(5):e0232723. doi: 10.1371/journal.pone.0232723 (PMC7241772; doi:10.1371/journal.pone.0232723)
Supplement: S2 Table — (DOCX) [file pone.0232723.s002.docx]

**S2 Table. Tests of deviation from the HWE for each locus and sampling group combination.**

Displayed are the P-values from the Fisher’s exact test.
