# Supplementary material for: Restored river habitat provides a natural spawning area for a critically endangered landlocked Atlantic salmon population
Source: PLoS One. 2020 May 21;15(5):e0232723. doi: 10.1371/journal.pone.0232723 (PMC7241772; doi:10.1371/journal.pone.0232723)
Supplement: S3 Table — (DOCX) [file pone.0232723.s003.docx]

**S3 Table. Consistency of the estimates of effective population size.**

Estimates of effective population size (Ne) with their associated 95% confidence intervals (Ne 95% CI) from nine different runs – three replicates for each combination of assumed errors – for each yearly cohort of parents. Means with their variance for each yearly cohort are shown in bold.
